# Construction of a genetic linkage map and QTL analysis of erucic acid content and glucosinolate components in yellow mustard (*Sinapis alba* L.)

**DOI:** 10.1186/1471-2229-13-142

**Published:** 2013-09-26

**Authors:** Farzad Javidfar, Bifang Cheng

**Affiliations:** 1 Agriculture and Agri-Food Canada, Saskatoon Research Centre, 107 Science Place, S7N 0X2 Saskatoon, SK, Canada

**Keywords:** Yellow mustard, ILP marker, Linkage map, QTL analysis, Glucosinolate

## Abstract

**Background:**

Yellow mustard (*Sinapis alba* L.) is an important condiment crop for the spice trade in the world. It has lagged behind oilseed *Brassica* species in molecular marker development and application. Intron length polymorphism (ILP) markers are highly polymorphic, co-dominant and cost-effective. The cross-species applicability of ILP markers from *Brassica* species and *Arabidopsis* makes them possible to be used for genetic linkage mapping and further QTL analysis of agronomic traits in yellow mustard.

**Results:**

A total of 250 ILP and 14 SSR markers were mapped on 12 linkage groups and designated as Sal01-12 in yellow mustard. The constructed map covered a total genetic length of 890.4 cM with an average marker interval of 3.3 cM. The QTL for erucic content co-localized with the *fatty acid elongase 1* (*FAE1*) gene on Sal03. The self-(in)compatibility gene was assigned to Sal08. The 4-hydroxybenzyl, 3-indolylmethyl and 4-hydroxy-3-indolylmethyl glucosinolate contents were each controlled by one major QTL, all of which were located on Sal02. Two QTLs, accounting for the respective 20.4% and 19.2% of the total variation of 2-hydroxy-3-butenyl glucosinolate content, were identified and mapped to Sal02 and Sal11. Comparative synteny analysis revealed that yellow mustard was phylogenetically related to *Arabidopsis thaliana* and had undergone extensive chromosomal rearrangements during speciation.

**Conclusion:**

The linkage map based on ILP and SSR markers was constructed and used for QTL analysis of seed quality traits in yellow mustard. The markers tightly linked with the genes for different glucosinolate components will be used for marker-assisted selection and map-based cloning. The ILP markers and linkage map provide useful molecular tools for yellow mustard breeding.

## Background

Yellow mustard (*Sinapis alba* L; genome SS, 2n = 24) is an obligate out-crossing crop due to its self-incompatibilty reproduction system. It is more heat and drought tolerant, and more resistant to pod shattering and diseases such as blackleg than *Brassica napus* and *B. rapa*[1-3]. Yellow mustard is well adapted to the semi-arid areas of western Canada and has been cultivated as a condiment crop in the Prairies since 1936 [4]. Condiment yellow mustard varieties contain a desirable high 4-hydroxybenzyl (HBEN) glucosinolate (GSL) component in the seed [3], which hydrolyses to produce the spicy "heat" sensation in the mouth. In addition, yellow mustard contains 3-indolylmethyl (IND), 4-hydroxy-3-indolylmethyl (HIND) and 2-hydroxy-3-butenyl (HBUT) GSL components in the seed.

Genetic linkage mapping has proven to be very useful for analyzing quantitative trait loci (QTL), tagging and cloning genes controlling desirable agronomic traits and studying genome organization and evolution. Construction of genetic linkage maps based on various molecular markers has revealed the occurrence of large-scale duplication as well as extensive chromosomal rearrangement in *B. rapa*[5,6] and *B. oleracea*[7], *B. napus*[8,9] and *B. juncea*[10,11]. QTL analysis identified two major QTLs for erucic acid content in *B. napus* and *B. juncea*[12,13]. Five QTLs for total glucosinolate content were mapped on chromosomes A2, A9, C2, C7, and C9 in *B. napus*[14,15]. Mahmood et al. [16] identified five QTLs explaining approximately 30 to 45% of the total aliphatic glucosinolate content variation in *B. juncea*.

Yellow mustard has lagged far behind oilseed *Brassica* species in molecular marker development and application. This could be due to the following reasons. Firstly, yellow mustard has a sporophytic self-incompatibility reproductive system which makes it difficult to develop homozygote parental and recombinant inbred lines required for linkage mapping. Secondly, yellow mustard is not a major food crop and therefore doesn’t receive much attention and funding for genomic research. So far, only one linkage map based on restriction fragment length polymorphism (RFLP) markers was constructed using populations derived from heterozygous parental lines in yellow mustard [17]. However, application of RFLP marker technology in genetic research and breeding is limited due to the laborious procedures and high cost. Single nucleotide polymorphism (SNP) and simple sequence repeat (SSR) markers have proven to be very useful for construction of high density maps in *B. napus* and *B. juncea*. However, these markers have not been developed in yellow mustard yet.

Intron length polymorphism (ILP) markers are highly polymorphic, co-dominant, cost-effective and cross-species applicable [9,18]. ILP primers in *B. napus*, *B. rapa* and *A. thaliana* have been developed [10,18]. Yellow mustard, *B. napus* and *B. rapa* belong in the Subtribe Brassicinae [19], and might have evolved from the same ancestor species as *A. thaliana*[10,20-22]. Therefore, the ILP primers from *Brassica* species and *Arabidopsis* could be used for genetic linkage mapping in yellow mustard. Doubled-haploid (DH) and inbred lines have lately been produced in yellow mustard at Agriculture and Agri-Food Canada-Saskatoon Research Centre (AAFC-SRC) [23,24]. Molecular markers for the *fatty acid elongase 1* (*FAE1*) and self-(in) compatibility genes have also been developed in our lab [25]. The objectives of the present study were 1) to construct a genetic linkage map based on ILP and SSR markers using the F_2_ population derived from homozygote parental lines, 2) to identify QTLs for erucic acid content and GSL components, and 3) to assign the *FAE1* and self-(in) compatibility genes to the respective linkage groups in yellow mustard.

## Results

### Polymorphism between the parental lines Y514 and Y517

A total of 1726 ILP primers – 383 from *A. thaliana*[10], 1093 from *B. napus* and 250 from *B. rapa* available in the Potential Intron Polymorphism (PIP) database [18] and 222 SSR markers with 73 from *B. napus* and 149 from *B. juncea* were used to screen the parental lines Y514 and Y517 for polymorphic markers. Of the 1726 ILP primers, 230 (13.3%) generated clear and scorable polymorphic bands between the parental lines varying in size from 100 bp to 1300 bp. Amongst the 230 polymorphic primers, 211 (91.7%) each amplified DNA fragments from one locus and 18 primers (7.8%) each revealed two loci while the remaining one (0.5%) revealed three polymorphic loci. To sum up, a total of 250 polymorphic loci were scored including 141 co-dominant and 109 dominant markers. In addition to the polymorphic loci, 253 monomorphic bands were amplified by 146 primers. Taking into account both polymorphic and monomorphic bands, a total of 503 loci were detected by the 230 ILP primers with an average of 2.2 loci/ILP primer. Only 14 (6.3%) out of the 222 SSR primers amplified polymorphic fragments between the parental lines, which comprised 5 (35.7%) co-dominant and 9 (64.3%) dominant loci. The 250 ILP and 14 SSR polymorphic markers were used to construct the linkage map with the F_2_ population of Y514 × Y517 in yellow mustard.

### Construction of a genetic linkage map

**Figure 1:**
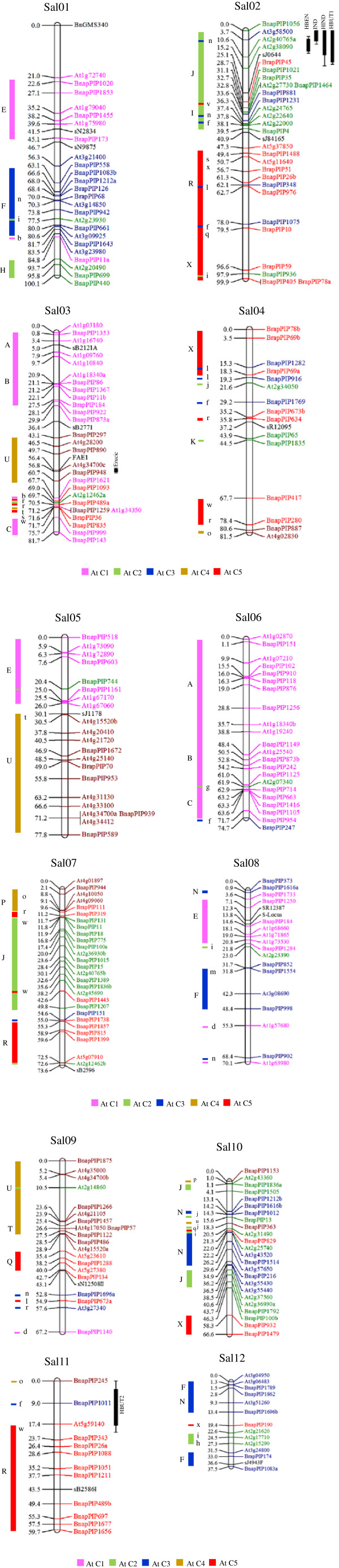
**The 12 linkage groups (Sal01-12) in yellow mustard.** For each linkage group, the ILP and SSR markers were shown on the right side, and the marker position in centiMorgan on the left side. The font colours of the markers and the colours of the highlighted conserved blocks indicated their chromosomal origin in *A. thaliana*. For each conserved block, the name was indicated on the left side and scattered markers were on the right side. The QTL for erucic acid content (ERU) co-localized with the *FAE1* gene on Sal03. The QTLs for HBEN, IND, HIND and HBUT were mapped to Sal02. The other QTL for HBUT was mapped to Sal11. 1-LOD and 2-LOD supporting intervals of each QTL were marked by thick and thin bars, respectively.

The 264 polymorphic loci between Y514 and Y517 were mapped on 12 linkage groups and covered a genetic length of 890.4 CentiMorgans (cM) (Table 1, Figure 1). The map length of the 12 linkage groups ranged from 37.5 to 100.1 cM with an average marker interval of 3.3 cM. They were designated as Sal01 to Sal12 in descending order of the genetic length. Sal01 and Sal02 had a similar long map length and average marker interval. However, Sal01 had one unmapped island (21.0 cM gap) located between the two markers BnGMS340 and At1g72740. Sal04 was similar with Sal03 in map length, but had the largest average marker interval (5.1 cM) and one unmapped island (23.2 cM gap) located between the two markers BnapPIP1835 and BnapPIP417. Sal05, Sal06, Sal07 and Sal08 had similar map length ranging from 77.8 cM to 70.1 cM. Sal07 had the smallest average marker interval of 2.6 cM. Sal09 and Sal10 were similar in both map length and number of markers. Sal11 had a map length of 59.7 cM with an average marker interval of 4.6 cM. The shortest linkage group Sal12 had 13 ILP and 1 SSR markers and a small average marker interval of 2.7 cM. The *FAE1* gene was located adjacent to the marker At4g34700c on Sal03 with a genetic distance of 0.2 cM. The self-(in)compatibility gene was located close to the marker BnapPIP184 on Sal08 with a genetic distance of 0.8 cM. The ILP markers were evenly distributed on the 12 linkage groups which likely represented the 12 chromosomes in yellow mustard.

**Table 1:** Characterization of the 12 linkage groups in yellow mustard

**Linkage group**	**Map length (cM)**	**Marker interval (cM)**			**No. of markers**		
		**Average**	**Max distance (cM)**	**Min distance (cM)**	**ILP**	**SSR**	**Total**
Sal01	100.1	3.7	21.0	0.3	24	3	27
Sal02	99.9	3.3	17.1	0.0	28	2	30
Sal03	81.7	2.8	11.2	0.0	27	2	29
Sal04	81.5	5.1	23.2	0.6	15	1	16
Sal05	77.8	3.5	12.8	0.1	21	1	22
Sal06	74.7	3.4	9.8	0.1	22	0	22
Sal07	73.6	2.6	12.9	0.1	27	1	28
Sal08	70.1	3.9	13.1	0.1	17	1	18
Sal09	67.2	3.2	13.1	0.0	20	1	21
Sal10	66.6	2.8	12.0	0.1	24	0	24
Sal11	59.7	4.6	8.4	2.2	12	1	13
Sal12	37.5	2.7	6.5	0.2	13	1	14

The majority (83.1%) of the 264 mapped markers segregated with the expected 1:2:1 or 3:1 Mendelian ratios. However, 16.9% markers deviated from the expected segregation ratio of 1:2:1 or 3:1 (0.01 ≤ P ≤ 0.05). These distorted markers were unevenly distributed on linkage groups Sal03, Sal06, Sal08 and Sal10. Among the distorted marker loci, 29 (64.4%) loci skewed towards the homozygous Y517 genotype whereas 11 (24.4%) loci skewed towards the homozygous Y514 genotype. The remaining 5 loci (11.1%) skewed towards the heterozygous genotype. Interestingly, DNA fragments not observed in the parental lines were generated in the F_1_ hybrid plants by 59 ILP primers. The new bands observed in the F_1_ plants appeared in the heterozygote F_2_ plants.

### Inheritance of HBEN, IND, HIND and HBUT GSL contents

**Figure 2:**
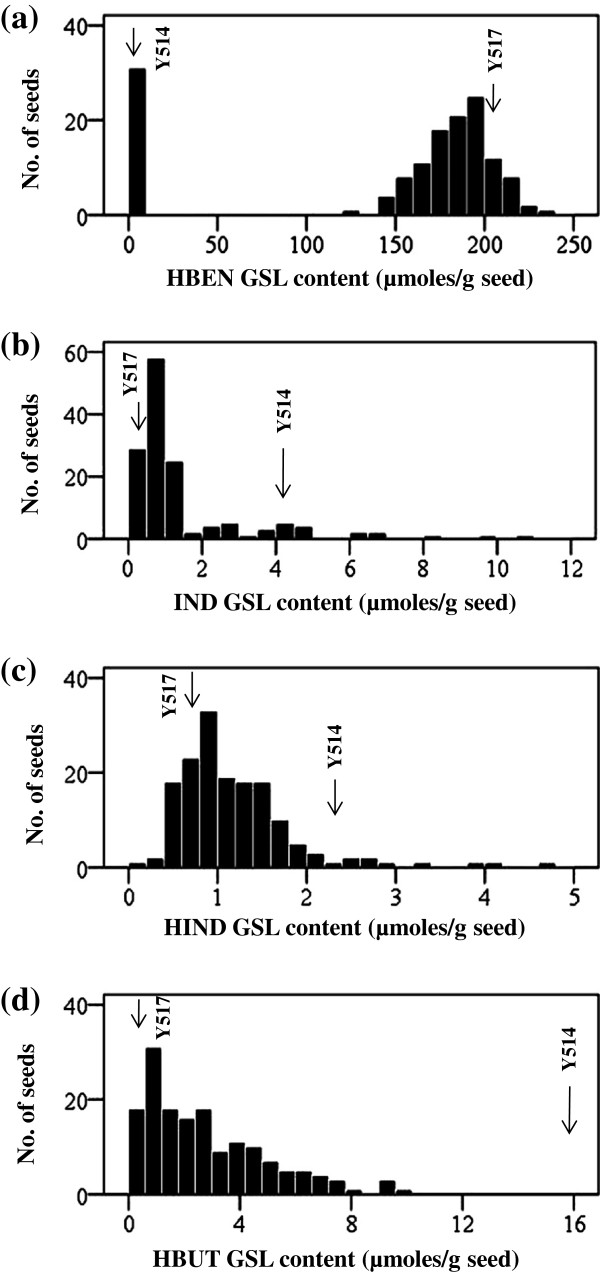
**Frequency distribution of HBEN (2a), IND (2b), HIND (2c) and HBUT (2d) glucosinolate contents of F**_**2 **_**plants of Y514 × Y517.** The arrows indicate the contents of the parental lines.

The F_1_ seed had similar HBEN, IND, HIND and HBUT GSL contents as the selfed female parental seed (Table 2), indicating that they were controlled by the maternal genotype. The F_3_ seeds borne on F_2_ plants were classified into two groups: seeds with zero (<0.3 μmoles/g seed), and seeds with medium to high (124.8-237.5 μmoles/g seed) HBEN contents, fitting well with a phenotypic ratio of 1 (zero):3 (medium to high) (χ^2^ = 1.0, P = 0.31) (Figure 2a). This result suggested that HBEN GSL was controlled by one gene locus with the dominance of high over the low content. The segregation of IND GSL content fitted with a phenotypic ratio of 3 (seeds with low content (0.2-1.6 μmoles/g seed)):1 (seeds with medium to high content (2.0-10.6 μmoles/g seed)) (Figure 2b), and was therefore under monogenic control with the dominance of low over the high content. HIND and HBUT contents of the F_3_ seeds exhibited continuous distribution and could not be classified into discrete groups (Figure 2c-d).

**Table 2:** **Erucic acid content, HBEN, IND, HIND and HBUT glucosinolate contents of parental lines Y514, Y517 and their reciprocal crosses F**_**1 **_**seeds**

**Genotype**	**Erucic acid**^**a **^**(%)**	**Glucosinolate (μmoles/g seed)**			
		**HBEN**	**IND**	**HIND**	**HBUT**
Parenal lines					
Y514	0.1 ± 0.0	0.1	4.3	2.3	16.3
Y517	52.9 ± 2.6	210.4	0.5	0.8	1.0
F_1_					
Y514 × Y517	34.6 ± 1.4	0.1	4.4	3.3	8.2
Y517 × Y514		217.0	0.5	0.7	0.6

^a^Erucic acid expressed as mean value ± standard deviation.

### QTL analysis of erucic acid content, HBEN, IND, HIND and HBUT GSL contents

QTL analysis was performed for erucic acid content, HBEN, IND, HIND and HBUT contents (Table 3). One QTL (LOD = 83.5), accounting for 92.3% of erucic content variation, was detected and co-localized with the *FAE1* gene on Sal03 (Figure 1). One QTL (LOD = 83.1), explaining 93.1% of the HBEN content variation was assigned to Sal02. It was located between the two markers At3g58500 and At2g40765a (Figure 1). One QTL (LOD = 36.1) explained 68.8% of the phenotypic variation of IND and was mapped in the region between the two markers BnapPIP1056 and At3g58500 on Sal02. One QTL (LOD = 13.4) for HIND was detected and located in the vicinity of the marker At3g58500 on Sal02, which accounted for 35.1% of the total variation. Two QTLs for HBUT content were detected. The first QTL (LOD = 7.1) accounted for 20.4% of the total variation and was mapped to the region near the marker At2g40765a on Sal02. The second QTL (LOD = 6.5) for HBUT explained 19.2% of the total variation and was located adjacent to the marker BnapPIP1011on Sal11. The QTLs for HBEN, IND and HIND GSL contents as well as one of the two QTLs for HBUT content were mapped to a terminal region on the same linkage group Sal02 (Figure 1).

**Table 3:** **Summary of QTLs for erucic content, HBEN, IND, HIND and HBUT glucosinolate contents in the F**_**2 **_**population derived from Y514 × Y517**

**Trait**	**Linkage group**	**Marker or interval**	**Position cM**	**LOD score**	**LOD threshold**	**R**^**2 a**^
Erucic acid	Sal03	*FAE1*	56.4	83.5	3.8	92.3
HBEN	Sal02	At3g58500-At2g40765a	5.7	83.1	3.6	93.1
IND	Sal02	BnapPIP1056	0.0	36.1	3.6	68.8
HIND	Sal02	At3g58500	3.7	13.4	3.5	35.1
HBUT	Sal02	At2g40765a	10.6	7.1	3.5	20.4
	Sal11	BnapPIP1011	9.0	6.5	3.5	19.2

^a^Percentage of the total phenotypic variation explained by QTL.

### Synteny relationships between yellow mustard and *A. thaliana*

ILP markers were used for comparative analysis of the linkage maps of *S. alba* and *A. thaliana* (Figure 1, Table 4). All linkage groups of yellow mustard were chimeric in relation to *A. thaliana* chromosomes. Sal01 and Sal08 had markers of *A. thaliana* chromosome (AtC) 1, AtC2 and AtC3, which corresponded to three different conserved synteny blocks. Sal02 had four conserved blocks with two from AtC2 and another two from AtC5. Sal03 comprised 4 conserved blocks with three from AtC1 and one from AtC4. Sal04, Sal07 and Sal10 contained markers of AtC2, C3, C4 and C5, which were grouped into two conserved blocks in Sal04, and three conserved blocks in Sal07 and Sal10. Sal05 contained a large conserved block of AtC4 with 12 markers covering a genetic length of 40 cM (51.4% of the total map length), and one conserved block of AtC1 (Figure 1). Sal06 mainly contained marker loci of AtC1 whereas Sal11 had majority of markers of AtC5. Sal09 was the most chimeric linkage group with marker loci from all the five *A. thaliana* chromosomes (Figure 1). Sal12 carried three conserved blocks of AtC3. In addition to the conserved blocks, it was also observed that some markers of one At chromosome were scattered in a conserved block of a different At chromosome in the linkage groups of yellow mustard.

**Table 4:** **Summary of conserved blocks of *A. thaliana *in yellow mustard genome**

**Linkage group**	**AKB**^**a**^	**No. of loci**	**Length of block (cM)**	***A. thaliana *chromosome**
Sal01	E	6	24.1	1
Sal01	F	12	27.2	3
Sal01	H	3	6.4	2
Sal02	J	7	32.8	2
Sal02	I	4	2.1	2
Sal02	R	4	12.0	5
Sal02	X	3	3.3	5
Sal03	A	8	21.2	1
Sal03	B	4	7.8	1
Sal03	U	5	17.6	4
Sal03	C	2	6.0	1
Sal04	X	3	18.3	5
Sal04	K	2	0.6	2
Sal05	E	6	26.0	1
Sal05	U	12	40.0	4
Sal06	A	10	38.8	1
Sal06	B	5	12.6	1
Sal06	C	4	8.8	1
Sal07	P	2	0.5	4
Sal07	J	11	38.1	2
Sal07	R	5	17.5	5
Sal08	N	2	0.9	3
Sal08	E	7	18.2	1
Sal08	F	2	6.1	3
Sal09	U	8	22.3	4
Sal09	T	2	0.4	4
Sal09	Q	3	4.6	5
Sal10	J	3	3.1	2
Sal10	N	3	1.2	3
Sal10	N	5	10.3	3
Sal10	J	4	7.8	2
Sal10	X	2	8.3	5
Sal11	R	9	36.0	5
Sal12	F	4	2.8	3
Sal12	N	2	4.1	3
Sal12	F	3	6.0	3

^a^Ancestral karyotype blocks proposed by Schranz et al. [46].

## Discussion

Doubled haploid and highly inbred lines of yellow mustard were successfully produced in our lab. This allowed us to construct a genetic linkage map by using an F_2_ population derived from homozygote parents in this out-crossing crop species for the first time. ILP primers were designed based on the conserved exon sequence flanking the introns to exploit intron polymorphism. Therefore, each ILP marker locus revealed by any particular primer would likely represent a gene copy in the genome. Taking both polymorphic and monomophic loci into account, 50.4% of the 230 ILP primers in the present study revealed duplicated or triplicated loci, thereby suggesting that yellow mustard is a secondary polyploidy species. This is in agreement with the previous RFLP mapping results [26]. The twelve linkage groups most likely represented the twelve chromosomes in yellow mustard. The presence of the unmapped islands in linkage groups Sal01 and Sal04 could be due to the low polymorphism in the two regions between the parental lines. The possible reasons for the occurrence of distorted ILP loci could be related to the linkage of the markers with self-incompatibility and also to natural selection for the heterozygous genotypes with higher vigour than the homozygotes. Distorted segregation ratio of markers were reported in linkage mapping of *B. napus*[27] and *B. carinata*[28]. In the F_1_ hybrid, some ILP primer pairs generated new DNA fragments that were absent in the parents, suggesting the occurrence of extensive divergence of either side of the flanking exon sequences in the parental lines. Each of the two parental lines might only have flanking exon sequence complementary to either the forward or reverse primer, thereby leading to no amplification. However, the F_1_ plants derived from such two parental lines would contain flanking exon sites that could anneal with both forward and reverse primers and therefore produced a new band.

One QTL was detected for erucic content, which was in agreement with the monogenic control of this trait in previous reports [29,30]. The biosynthesis of erucic acid is controlled by the *FAE1* gene [31]. As expected, the QTL for erucic content co-localized with the *FAE1* gene on Sal03. The biosynthesis of glucosinolates occurs in the silique wall (maternal tissue) and is then transferred to the developing seeds [32]. Therefore, the HBEN, IND, HIND and HBUT contents were controlled by the maternal genotype. Genetic studies and QTL mapping indicated that HBEN content was controlled by one gene locus with dominance of the high over low content as reported by Drost et al. [33]. The two markers At3g58500 and At2g40765a were tightly linked with the HBEN content and could be used for marker-assisted selection and map-based cloning. The allele for high HBEN content was linked with the allele for low IND content with a recombination frequency of 5.7%. Of the two genes for HBUT, one had a recombination frequency of 4.9% with the gene for HBEN on Sal02, and the other one was located on the linkage group Sal11. These results indicate that it is possible to develop new germplasm with different GSL profile in yellow mustard.

HBEN is an aromatic GSL produced from tyrosine. IND and HIND are indole GSLs derived from tryptophan, and HBUT is an aliphatic GSL with methionine as the precursor [34]. In *A. thaliana*, the QTL controlling indolic GSL was not overlapped with QTL for aliphatic GSLs, but the major QTL controlling the accumulation of seed benzyl GSL is linked to the *GS-Elong* locus that controls total leaf aliphatic glucosinolates [35]. The present study revealed that the QTLs for HBEN, IND, HIND and one QTL of HBUT were overlapped with each other at 1-LOD confidence interval in Sal02. It remains to be investigated whether the four GSL QTLs located in the same region are controlled by the same gene or due to linkage of different genes for the various GSL components in yellow mustard.

Comparative synteny analysis revealed that the linkage groups of yellow mustard shared many conserved blocks with that of *A. thaliana*. In particular, Sal06 and Sal11 mainly had common marker loci with AtC1 and AtC5, respectively. This suggested that Sal06 and AtC1 could be derived from the same ancestor chromosome while Sal11 and AtC5 from different one. However, it was also observed that most linkage groups of yellow mustard shared markers with over three chromosomes of *A. thaliana*, implying that extensive structural rearrangements such as translocations involving various chromosomes of the ancestor species and inversions had occurred during the speciation of yellow mustard. This is in agreement with the previous mapping results based on RFLP markers in yellow mustard [36]. Comparative mapping of *Brassica* species and *A. thaliana* also revealed the occurrence of extensive segmental rearrangement in *B. nigra*[20], *B. napus*[37], *B. juncea*[10] and *B. oleracea*[38]. The occurrence of conserved blocks between *S. alba* and *A. thaliana* would enable the genomics knowledge transfer from this model species to yellow mustard.

The current cultivars in yellow mustard are heterogeneous population varieties. With the objective to develop high yielding synthetic varieties, elite inbred lines tolerant to inbreeding have been produced by purging the deleterious alleles in each inbred generation [24]. Characterization of the genetic diversity of different inbred lines is essential for selection of synthetic component lines with high heterotic potential. The ILP markers and constructed genetic linkage map in this study will greatly facilitate molecular assisted breeding in our yellow mustard program.

## Methods

### Plant material and production of mapping population

The quality profiles of the two parental lines Y514 and Y517 were shown in Table 2. Y514 was the DH line SaMD3 produced at AAFC-SRC [23]. It had a zero erucic content (average: 0.1%) and a zero HBEN content (average: 0.1 μmoles/g seed). Y517 was produced by seven generations of inbreeding of the F_1_ plant between the variety Sabre and the Svalöf high oil line (T. Olson, personal communication, 2010). It had a high erucic acid content (average: 52.9%) and a high HBEN content (average: 210.4 μmoles/g seed). In addition, Y514 and Y517 differed in IND, HIND and HBUT GSL contents (Table 2). Reciprocal crosses were made between Y514 and Y517 to produce the F_1_ seed. The F_1_ plants were self-pollinated to produce F_2_ seeds. One hundred fifty F_2_ plants from one F_1_ plant (Y514 × Y517) were used for constructing the genetic linkage map. The parental lines, F_1_ plants and F_2_ population were grown in the greenhouse at AAFC-SRC.

### DNA extraction and polymerase chain reaction (PCR)

Genomic DNA was extracted from young leaves of the parental, F_1_ and F_2_ plants using the modified sodium dodecyl sulfate method [39]. PCR for ILP markers was carried out according to Javidfar and Cheng [30]. For SSR marker analysis, the PCR mixture contained 50 ng genomic DNA, 200 μM of each dNTP, 1x PCR buffer, 200 nM of each primer and 1 U of Taq polymerase (New England Biolabs) in a final volume of 20 μl. The PCR was performed with an initial 5 min denaturation at 94°C; 20 cycles of 30 s at 94°C, 30 s at 56°C, 45 s at 72°C; 20 cycles of 30 s at 94°C, 30 s at 47°C, 45 s at 72°C; and a final 6 min extension at 72°C. Fragments were amplified with T7 fluorochrome- labeled primers (FAM, VIC, NED and PET) (Life Technologies). PCR products were combined and analyzed on a 3130xl DNA Analyzer with 600LIZ size standard (Life Technologies), and scored using GeneMapper 4 software (Life Technologies).

### Construction of genetic linkage map and QTL analysis

The genetic linkage map was constructed by using JoinMap version 4.0 at LOD scores ≥ 4.0 [40]. Recombination frequencies were converted to map distances in cM using the Kosambi mapping function [41] and the genetic map was drawn with MapChart [42]. Chi-square test for goodness-of-fit was performed to determine if marker segregation deviated from the expected ratio. The threshold of P < 0.01 was used to exclude the distorted markers from the map construction. An interval mapping analysis [43,44] was conducted using the MapQTL 6.0 software [45] to detect QTLs for erucic acid content, HBEN, IND, HIND and HBUT GSL contents. Permutation test (1,000 replications) was used to determine the significance level for LOD with a genome-wide probability of P < 0.05. An island was defined as a region with a gap of greater than 20 cM between two adjacent markers [10].

### Comparative synteny analysis

The constructed genetic linkage map in yellow mustard was compared with the established 24 genomic blocks (A-X) of *A. thaliana*[46]. A conserved block in the linkage map of yellow mustard was defined as a region with at least two closely linked ILP marker loci from the same block of *A. thaliana*.

### Mapping of the *FAE1* and self-(in)compatibility genes

Primer pair No 1 OF (ATGACGTCCGTTAACGTA) /PR (AAGACTTGTCGTCAGCTCCA) was designed based on the *FAE1* gene sequence of Y517 and generated a dominant marker of 928 bp for *FAE1* gene in Y517 (F. Zeng and B.F. Cheng, personal communication, 2012), while the primer pair No 2 Sal-SRK I (GATTATCTCGTGTCTGAATG/ GGTAATGTCGAATCTCTCCT) was designed based on the class I *S* haplotype of Y514 and produced a dominant marker of 640 bp for the self-(in) compatibility gene in Y514 [25]. The *FAE1* and self-(in)compatibility genes were mapped to their respective linkage groups based on the segregation of the two markers in the F_2_ population of Y514 × Y517. The PCR reaction mixture (20 μl) contained 1x PCR buffer, 1.5 mM MgCl_2_, 200 μM of each dNTP, 0.1 μM of each forward and reverse primer, 1 U of Taq polymerase (New England Biolabs) and 50 ng of genomic DNA. Polymerase chain reaction was performed with an initial denaturation at 94°C for 5 min followed by 30 cycles of 45 s at 94°C, 45 s at annealing temperature and 1 min at 72°C. A final extension cycle of 72°C for 5 min was conducted. All PCR products were analyzed by electrophoresis in 2% agarose gels in 1x TAE buffer. Gels were visualized by staining in 0.5 mg/L ethidium bromide and photographed on a digital gel documentation system.

### Erucic acid content and GSL profile assay

The erucic acid contents of the parental lines, F_1_ and F_2_ seeds were determined using the half-seed technique [47] and the gas chromatographic method of Thies [48], except that gas chromatography of the methyl esters was performed with a HP-INNOWax fused silica capillary column (0.25 mm × 7.5 m and 0.5 μm) (Agilent Technologies) at 250°C using hydrogen as the carrier gas. At least 20 seeds of each of the parental lines and 20 F_1_ seeds were half-seed analyzed for erucic acid content. Bulk samples of 30 self-pollinated seeds from each of the parental lines, 10 F_1_ seeds and 10 F_3_ seeds from each F_2_ plant were assayed for GSL profile using the method described by Raney et al. [49]. Benzyl GSL was isolated from the nasturtium seed in the chemistry lab at AAFC-SRC and used as standard.

## Conclusions

We have constructed a genetic linkage map with ILP and SSR markers and used it for QTL analysis of erucic acid content and glucosinolate components in yellow mustard. The markers tightly linked with the genes controlling different glucosinolate components will be used for marker-assisted selection and map-based cloning. The ILP markers and linkage map in this study provide useful molecular tools for yellow mustard breeding.

## Abbreviations

AAFC-SRC: Agriculture and Agri-Food Canada-Saskatoon Research Centre; AtC: Arabidopsis thaliana chromosome; cM: CentiMorgans; DH: Doubled-haploid; FAE1: *Fatty acid elongase 1*; GSL: Glucosinolate; HBEN: 4-hydroxybenzyl; HBUT: 2-hydroxy-3-butenyl; HIND: 4-hydroxy-3-indolylmethyl; ILP: Intron length polymorphism; IND: 3-indolylmethyl; PCR: Polymerase chain reaction; PIP: Potential intron polymorphism; QTL: Quantitative trait loci; RFLP: Restriction fragment length polymorphism; SNP: Single nucleotide polymorphism; SSR: Simple sequence repeat.

## Competing interests

The authors declare that they do not have competing interests.

## Authors’ contributions

BC conceived the project. FJ performed research and analyzed data. FJ and BC wrote the article. Both authors read and approved the final manuscript.
